# Fast 3D Rotation Estimation of Fruits Using Spheroid Models

**DOI:** 10.3390/s21062232

**Published:** 2021-03-23

**Authors:** Antonio Albiol, Alberto Albiol, Carlos Sánchez de Merás

**Affiliations:** 1 ITEAM Research Institute, Universitat Politècnica de València, 46022 Valencia, Spain; alalbiol@iteam.upv.es; 2 MultiScan Technologies S.L., 03820 Cocentaina, Spain; csanchez@multiscan.eu

**Keywords:** food inspection, rotation estimation, geometric modeling, real time, 3D, computer vision, image processing, image analysis

## Abstract

Automated fruit inspection using cameras involves the analysis of a collection of views of the same fruit obtained by rotating a fruit while it is transported. Conventionally, each view is analyzed independently. However, in order to get a global score of the fruit quality, it is necessary to *match* the defects between adjacent views to prevent counting them more than once and assert that the whole surface has been examined. To accomplish this goal, this paper estimates the 3D rotation undergone by the fruit using a single camera. A 3D model of the fruit geometry is needed to estimate the rotation. This paper proposes to model the fruit shape as a 3D spheroid. The spheroid size and pose in each view is estimated from the silhouettes of all views. Once the geometric model has been fitted, a single 3D rotation for each view transition is estimated. Once all rotations have been estimated, it is possible to use them to *propagate* defects to neighbor views or to even build a *topographic map* of the whole fruit surface, thus opening the possibility to analyze a single image (the map) instead of a collection of individual views. A large effort was made to make this method as fast as possible. Execution times are under 0.5 ms to estimate each 3D rotation on a standard I7 CPU using a single core.

## 1. Introduction

Food inspection is essential to ensure quality and safety in the food industry [1]. Many different techniques have been proposed in the literature to this end. Some of the most common methods use one of the following characteristics: optical properties, sonic vibration, computer vision, nuclear magnetic resonance (NMR), electronic noses, electrical properties, and computed tomography [2].

Among these techniques, computer vision has become a standard solution for food inspection [3,4] because it is one of the most economic and fastest options available [5]. Computer vision can be used to assess external appearance factors such as the the size, shape, color, and texture [6].

One of the applications of machine vision, and the main reason that motivated this work, is the capacity of computer vision to detect skin defects in fruits, such as insect attacks or rotten portions [7]. However, to achieve this goal, it is necessary to obtain images of the whole surface of the fruit. This is usually accomplished by capturing multiple overlapping views of each fruit in industrial inspection machines. Still, two problems remain open:

- Many views of the fruit do not guarantee that the whole surface has been observed. Therefore, a method is needed to assess which fraction has been viewed.
- To prevent multiple counting of defects, it is necessary to match points in different views.

**Figure 1:**
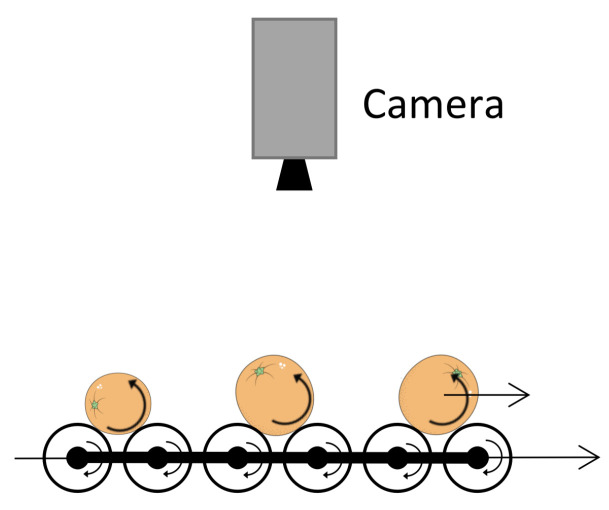
Roller conveyor unit used to obtain different views of the rotated fruits.

In this work, a roller conveyor unit is used to obtain different rotated views of each fruit as they travel under the camera as it is shown in Figure 1. The rotation speed can be adjusted independently of the linear traveling speed.

The objective of this work is to estimate the 3D rotations between pairs of consecutive views of rotating fruits so that surface defects can be tracked. The estimated rotations can also be used to evaluate which portion of the whole surface has been observed.

Normally, controlled visible or infrared illuminations are used in this kind of machines so that segmentation and tracking of each fruit become a trivial problem using standard image processing techniques [8].

**Figure 2:**
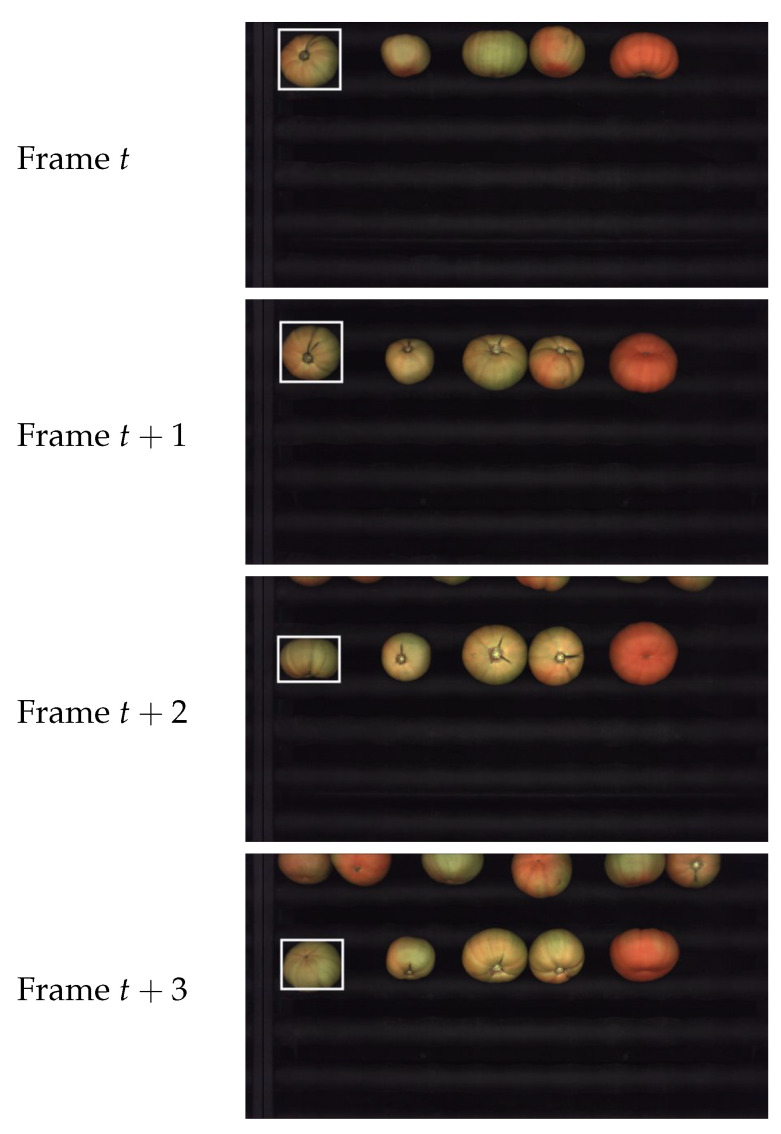
Four consecutive camera frames.

**Figure 3:**
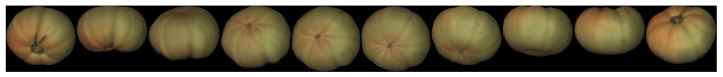
Set of views of the fruit highlighted in Figure 2.

Figure 2 shows a few consecutive frames captured by the camera. The same fruit is highlighted in all the images to illustrate how it rotates while moving downwards. After segmentation, it is possible to obtain a set of views for each fruit, as it is shown in Figure 3 for the tomato highlighted in Figure 2.

3D rotations can only be applied to 3D objects, and, for this reason, a geometric model for the fruits is needed. In this work, it is assumed that the shape of the fruits can be modeled by a 3D spheroid as a first approximation. For this reason, fruits like pears, eggplant, cucumbers, bananas, etc. are not adequate for the proposed method and are out of the scope of this research.

The general idea of how the 3D rotations are obtained is conceptually simple once the 3D models are fitted to the fruits. In short, a number of candidate 3D rotations are tried for the source fruit, and the one that minimizes a cost function is selected. This happens when the transformed source fruit is most similar to the target fruit. This procedure is quite similar to how motion is estimated for each block in block-matching [9] where several 2D displacements are also tried for each block.

**Figure 4:**
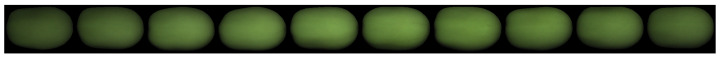
Example of green tomato with no texture. No 3D motion can be estimated in this case.

An important limitation of motion estimation is that the moving object must have some texture. For instance, if a fruit has a perfectly smooth and uniform skin (Figure 4), it will be impossible to obtain the rotation even by a human observer. In our tests, we only had this problem with some varieties of tomatoes, where the proposed methodology can not work.

One key aspect when designing algorithms for industrial applications is efficiency. Industrial inspection machines require high throughput and very often conventional PC hardware for image analysis is used. In block matching, all the pixels within a block undergo the same 2D displacement; however, the same does not happen for 3D rotations. In this case, the projected displacement of a pixel on the image, for a given 3D rotation, is different for each pixel and must be calculated. Unfortunately, rotating all the source fruit pixels is computationally too expensive. Therefore, it is necessary to use a few tricks to keep the computational burden low. Using these tricks, computational times on the order of 0.5–1 ms/view for the whole process on a 2018 I7-based PC using one single core are achieved. Considering around 10–12 views per fruit, this will allow for estimating the rotations of about 80 fruits per second. In the case of oranges or large tomatoes (about 200 g. per fruit), these numbers translate into a theoretical throughput of over 50 tons/h of product.

The main contribution of this work is a novel method to estimate 3D fruit rotations using the same camera present in vision based industrial inspection machines. This method can be used to prevent multiple detections of the same skin defect and assess what percentage of the fruit surface has been viewed. These goals can be accomplished without introducing any hardware change (additional acquisition equipment) in existing industrial roller inspection conveyors; only software changes are required. Moreover, the low computational cost required by the algorithm allows its integration in the same machine together with the rest of the image analysis functions.

Alternative approaches to this problem (see Section 2) rely on the use of multiple cameras, depth-cameras, or robotic arms that imply major modifications of current existing industrial machines.

## 2. Related Work

Detection of skin defects in fruits requires that the whole surface of each fruit is imaged. Current solutions to this problem can be broadly classified into three groups:

- Capturing multiple images from different views by using multiple cameras.
- Using a single camera with several helping mirrors.
- Rotating the fruits using rollers or robot hands.

Each of these solutions offers advantages as well as drawbacks.

The use of multiple cameras is the most common used method in in-line inspection [10,11,12,13]. In [14], three cameras are used to scan whole surface of apples. Defects are counted in each of the views, and the fruit is accepted or rejected based on this count. Although this strategy is effective, cost of cameras, synchronization, and complexity are important practical issues to be considered. In addition, false rejections may occur if defects are counted multiple times on the overlapping views.

An interesting alternative for capturing the whole fruit surface is to use mirrors so that the fruits are viewed from multiple view angles. In [15], two mirrors on opposite sides of an apple are used to capture much of the surface, although the supporting mechanism blocked some parts, and the processing speed of 3–4 apples per second is lower than required commercial speeds. The use of mirrors was extended in [16] to also measure the 3D shape of strawberries. A comprehensive study on the use of mirrors to reconstruct the whole surface of fruits can be found in [17], where different configurations of concave and flat mirrors are compared and different configurations with two, four, and six mirrors are also explored. The study concludes that shape distortions in reflected images and duplicated parts in multiple views are adverse issues of these approaches. Another important practical disadvantage of these methods is the dirt accumulation on the mirrors [18] and the difficulty to scan several fruits in parallel as in Figure 2.

The whole surface of the fruits can also be imaged by rotating the fruits. In [19], a robotic grading system was developed for several fruit types. The system was able to capture multiple images of the inspected fruits while they were sucked up by rotating suction pads. Other authors propose to control the rotation of each fruit [20]. For instance, in [21], the whole surface of mango fruits was captured using four images after rotating the fruit 90° between each acquisition. However, precise rotation of fruits is very slow and is not adequate for the high throughput required by industrial inspection.

In practice, the use of a roller conveyor is the the most common approach to rotate fruits [22,23]. However, the rotation is not well controlled due to differences in fruit sizes and shapes; therefore, some surface portions might be overlapped or missed due to the non-uniform rotation.

A common problem to all the above methods is how to match the different views so that defects are not counted more than once [7]. Surface reconstruction is one possible solution to this problem. In [24], the surface of fruits is reconstructed in 3D by using RGB-D cameras. However, the need of very specialized cameras that must operate at very high frame rates limits the applicability of this approach for existing machines.

The matching problem can also be solved if the 3D motion of the fruit between views is recovered. 3D motion estimation is a well studied problem, with many applications in very different fields. Early works on 3D motion estimation using a single camera used object projections [25,26]. However, the rotational symmetry of many of the fruits of interest makes this approach impractical. The 3D motion can also be recovered using a single camera if some constraints about the object shape are applied. For instance, in [27], objects are modeled using simple geometric primitives and the projections are linked with dual space geometry. Other examples of simple geometric primitives used in the literature include polyhedral models [28] and spheroid models [12].

## 3. Materials and Methods

### 3.1. Modeling the 3D Shape of the Fruits

In this paper, the 3D shape of a fruit is approximated using a spheroid, also known as ellipsoid of revolution.

**Figure 5:**
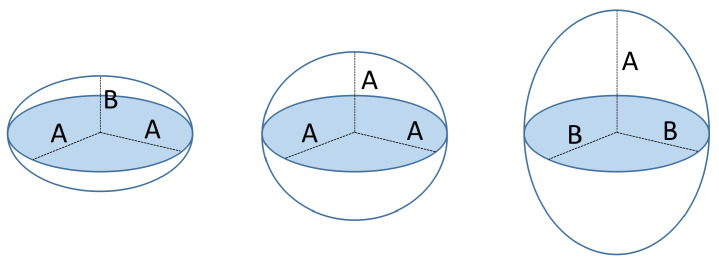
**Left**: oblate spheroid model; **Center**: sphere model; **Right**: prolate spheroid model.

**Figure 6:**
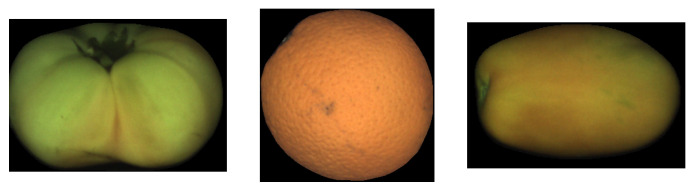
Samples of different fruit shapes. **Left**: oblate; **Center**: spherical; **Right**: prolate.

A spheroid is a particular kind of ellipsoid that has at least two equal principal axes. Depending on whether the different axis is shorter or longer than the equal ones, the ellipsoid is called *oblate* or *prolate*, respectively [29]. In the case that all the principal axes have the same length, the spheroid becomes a sphere. Figure 5 shows an example with the different spheroid types. Examples of fruits that approximate these shapes are also shown in Figure 6.

An interesting property of ellipsoids, and spheroids in particular, is that the shapes of their orthogonal projections are ellipses [30]. Given the size of the fruits and the typical height of the camera (about 1 m), perspective effects are negligible and a parallel camera can be assumed locally for each fruit [31]. Moreover, in the case of spheroids, the length of one of the principal axes of the projected ellipse is equal to one of the two equal principal axes of the spheroid. This property is used in Section 3.1.2 to determine the length of all the principal axes of the spheroid using all the available 2D views (Figure 3).

In this section, it is assumed that a binary mask indicates which pixels correspond to the fruit exists for each view. In practice, since the illumination conditions are controlled, this mask can be easily obtained by appropriately thresholding in the HSI colorspace. However, the details of this step are out of the scope of this paper and may be different depending on the fruit type.

Given a binary mask, it is possible to obtain the length of the principal axes of the projected ellipse, as it is detailed in Section 3.1.1. Using these values from all the available views, it is possible to infer the length of the principal axes of the spheroid, as described in Section 3.1.2. Finally, the fitting process ends by calculating the elevation angle and the 3D coordinates of all the fruit pixels as presented in Section 3.1.3 and Section 3.1.4, respectively.

#### 3.1.1. Principal Axes of the Projected Ellipses

**Figure 7:**
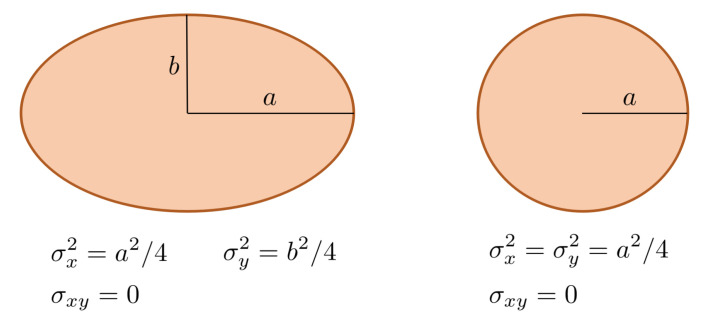
Relation of variances and semi-principal axes for an axis-aligned ellipse (circle).

Given a 2D axis-oriented ellipse (circle) shape, it is possible to relate the variances of its pixel coordinates to the lengths of the semi-principal axes (radius). These relations are depicted in Figure 7 and can be easily obtained assuming a 2D elliptical uniform distribution for the pixel coordinates and then calculating the second order moments: $\sigma_{x}^{2}$, $\sigma_{y}^{2}$ and $\sigma_{xy}\;$[32].

In the case that the ellipse is not axis-aligned, the relation is similar but using the eigenvalues of the covariance matrix.

Let Σ be the covariance matrix and $\lambda_{1}$ and $\lambda_{2}$ its corresponding eigenvalues ($\lambda_{1}\geq \lambda_{2}$):

$$\Sigma =\left(\begin{array}{cc} \sigma_{x}^{2} & \sigma_{xy}\\ \sigma_{xy} & \sigma_{y}^{2} \end{array}\right)$$

Then, the lengths of the semi-major and semi-minor principal axes are respectively:

(1) $$a=2\sqrt{\lambda_{1}}\;\;b=2\sqrt{\lambda_{2}}$$

**Figure 8:**
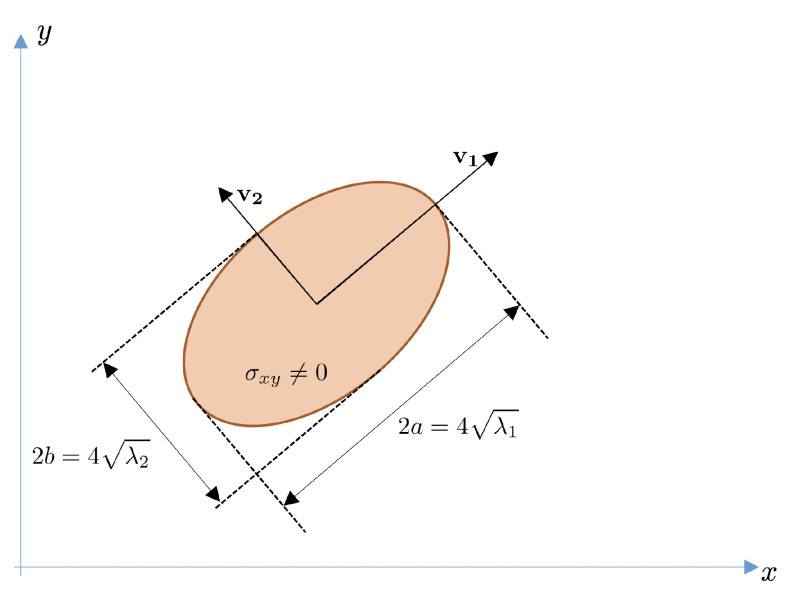
Relation between the variances and principal axes in the case of a rotated ellipse. $\lambda_{1}$ and $\lambda_{2}$ are the eigenvalues of the covariance matrix.

Figure 8 shows the relation between the length of the principal axes and the eigenvalues of Σ.

The covariance matrix Σ is estimated using the following expressions:

(2) $$S_{x}=\sum_{p=1}^{N}x_{p}\;\;S_{y}=\sum_{p=1}^{N}y_{p}$$

$$S_{xx}=\sum_{p=1}^{N}x_{p}^{2}\;\;S_{yy}=\sum_{p=1}^{N}y_{p}^{2}\;\;S_{xy}=\sum_{p=1}^{N}x_{p}\;y_{p}$$

where $\left(x_{p},y_{p}\right)$ are the coordinates of a pixel under the mask, and *N* is the total number of such pixels. Then, the variances and object center are estimated from the previous sums as:

(3) $$c_{x}=S_{x}/N\;\;c_{y}=S_{y}/N$$

$$\sigma_{x}^{2}=S_{xx}/N-c_{x}^{2}\;\;\sigma_{y}^{2}=S_{yy}/N-c_{y}^{2}\;\;\sigma_{xy}=S_{xy}/N-c_{x}\;c_{y}$$

One trick to accelerate the computation of moments is to use a stride greater than one when computing the sums of Equation (2). For instance, using a stride of 4 reduces the time of this part by a factor $4^{2}=16$. Our preliminary results showed that the error produced by using a stride larger than one is negligible and much less than the error caused because the shape of the binary masks is not perfectly elliptical.

#### 3.1.2. Determination of the Spheroid Principal Axes

This section explains how to estimate the three principal axes of the spheroid model using the major and minor principal axes of the projected ellipses from all the views. Depending on the spheroid type, the procedure slightly changes as described next. Since spheroids have at least two equal principal axes, there are two unknowns *A* and *B* which correspond to the lengths of the longest and shortest semi-principal axes, respectively (Figure 5). The number of available views for each fruit will be denoted as $N_{v}$.

##### Spherical Model

In this case, the three spheroid principal axes are identical (A=B) and the projected shape of the fruit will be a circle with the same radius as the sphere. However, since in practice the spherical shape is only an idealization, the radius of the sphere is obtained by using the mean of the semi-major and semi-minor principal axes from all the views:

(4) $$A=B=\frac{1}{N_{v}}\sum_{i=1}^{N_{v}}\frac{a_{i}+b_{i}}{2}$$

where $a_{i}$ and $b_{i}$ are the semi-major and semi-minor axes of the *i*-th view.

##### Oblate Model

**Figure 9:**
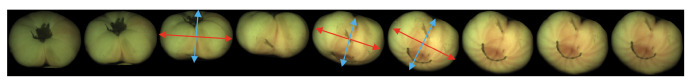
Views of an oblate fruit. The major principal axes are very similar in all views (2a≈2A). The range of the minor principal axis in each view is $2B<2b_{i}<2A$. The minor principal axis of the spheroid is visible in the fourth view starting from the left ($b_{4}\approx B$).

The orthogonal projection of a spheroid always allows for measuring the length of its equal principal axes on the projected ellipse. Therefore, for oblate spheroids, the length 2A of the equal principal axis is visible in all views. This is illustrated in Figure 9, where the major axes (in red) in all ellipses have a similar length. The shortest principal axis of the spheroid, *B*, will be observable only if it is orthogonal to the camera axis in at least one view.

Thus, the length of the semi-principal axes of the ellipsoid is estimated as:

(5) $$A=\frac{1}{N_{v}}\sum_{i=1}^{N_{v}}a_{i}\;\;B=\min_{i}b_{i}$$

Figure 9 gives an example for an oblate fruit, where the relation between the major axes of the projected ellipses and the major axes of the spheroid can be easily seen.

##### Prolate Model

Now, the length of the equal semi-principal axes of the spheroid is *B* and the longest principal axis of the spheroid *A* will be observable only if it is orthogonal to the camera axis in at least one view.

Therefore, the principal axes of the ellipsoid are estimated as:

(6) $$A=\max_{i}a_{i}\;\;B=\frac{1}{N_{v}}\sum_{i=1}^{N_{v}}b_{i}$$

#### 3.1.3. Elevation Angle Estimation

The goal of this section is to obtain the orientation of the 3D spheroid relative to the camera axis. This section applies only to non-spheric objects. The discussion below will be for oblate objects. A similar reasoning can be derived for prolate ones.

**Figure 10:**
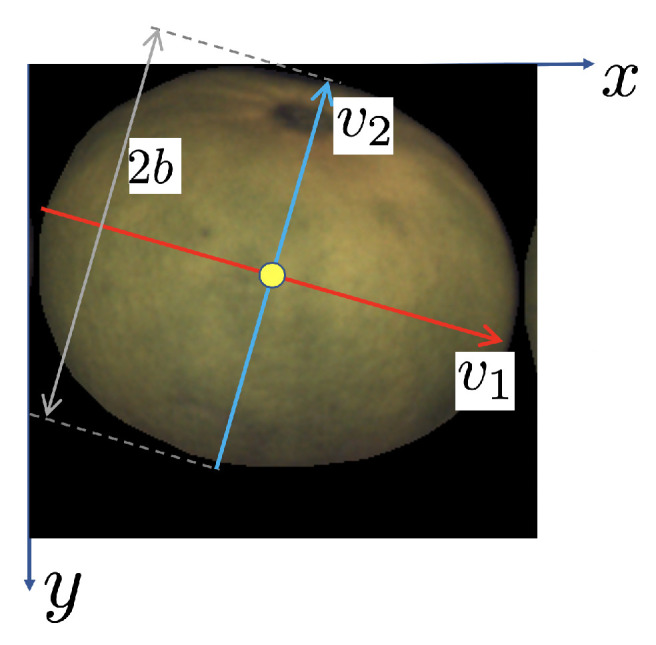
Sample camera view of an oblate object. The red axis is oriented as the eigenvector corresponding to the largest eigenvalue of Σ. Its length is the same as the major spheroid semi-axis, 2A. The yellow circle is located at the center of mass of the fruit/view.

Consider one view of an oblate object such as the one depicted in Figure 10. The *x*- and *y*-axis will correspond to the image axes. The *z*-axis is normal to the image. The $v_{1}$-axis is oriented as the eigenvector of Σ associated with its major eigenvalue, $\lambda_{1}$. The $v_{2}$-axis is orthogonal to $v_{1}$ and corresponds to the direction of the eigenvector associated with the minor eigenvalue, $\lambda_{2}$.

**Figure 11:**
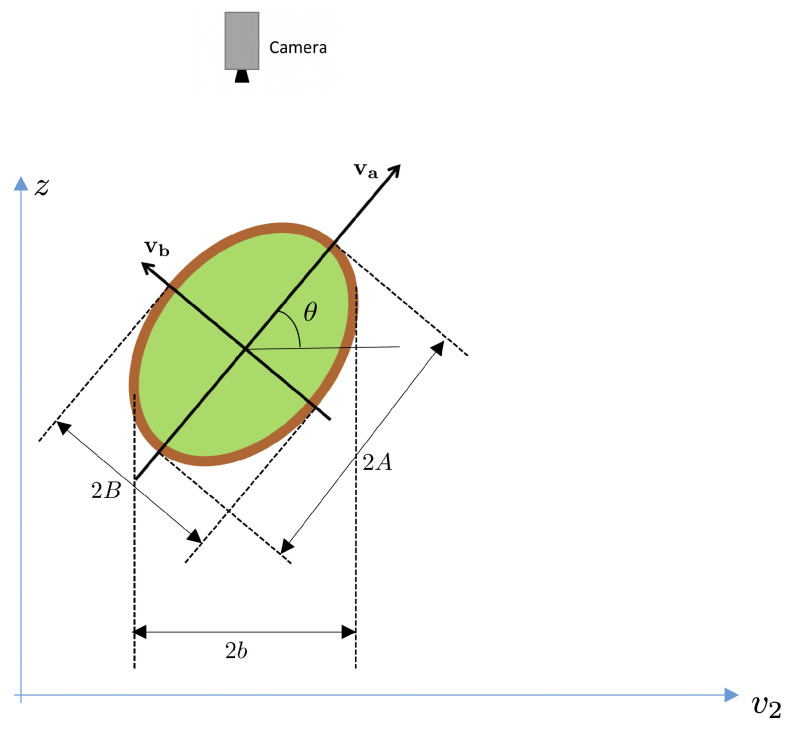
Cross section of fruit across 3D plane $v_{1}=0$ in Figure 8. Camera position above the fruit is shown.

Let’s consider a cross-section of the fruit in Figure 10 through the 3D plane $v_{1}=0$. This cross-section is shown in Figure 11. Notice that, in this figure, the axes are $v_{2}$ and *z* and allow for visualizing the principal spheroid axes ($v_{a}$ and $v_{b}$).

Then, the elevation angle θ is defined as the angle between the $v_{a}$ axis and the camera plane, as illustrated in the same figure. Notice from Figure 11 that the length of the observed minor axis on the image, 2b, depends on the spheroid dimensions (*A* and *B*), and the elevation angle θ.

In Section 3.1.1, it was shown that there exists a direct relation between the lengths of the principal axes of an ellipse and the covariance matrix Σ of the pixel coordinates. In [30], it is shown how to obtain the variances of the ellipsoid projections using its own variances.

The following relation between axes exists (Figure 11):

$$v_{2}=v_{a}\;\cos\theta -v_{b}\;\sin\theta$$

Computing the variance on both sides, we obtain the relation:

$$\lambda_{2}=\sigma_{A}^{2}\;\cos^{2}\theta +\sigma_{B}^{2}\sin^{2}\theta$$

where $\sigma_{A}^{2}=A^{2}/4$, $\sigma_{B}^{2}=B^{2}/4$ and $\lambda_{2}=b^{2}/4$ (recall that $\lambda_{2}$ is the variance along direction $v_{2}$).

Therefore, the following relation holds:

$$b^{2}=A^{2}\cos^{2}\theta +B^{2}\sin^{2}\theta =A^{2}\cos^{2}\theta +B^{2}\left(1-\cos^{2}\theta \right)$$

from which the angle θ can be isolated as:

(7) $$\cos\theta =\sqrt{\frac{b^{2}-B^{2}}{A^{2}-B^{2}}}$$

**Figure 12:**
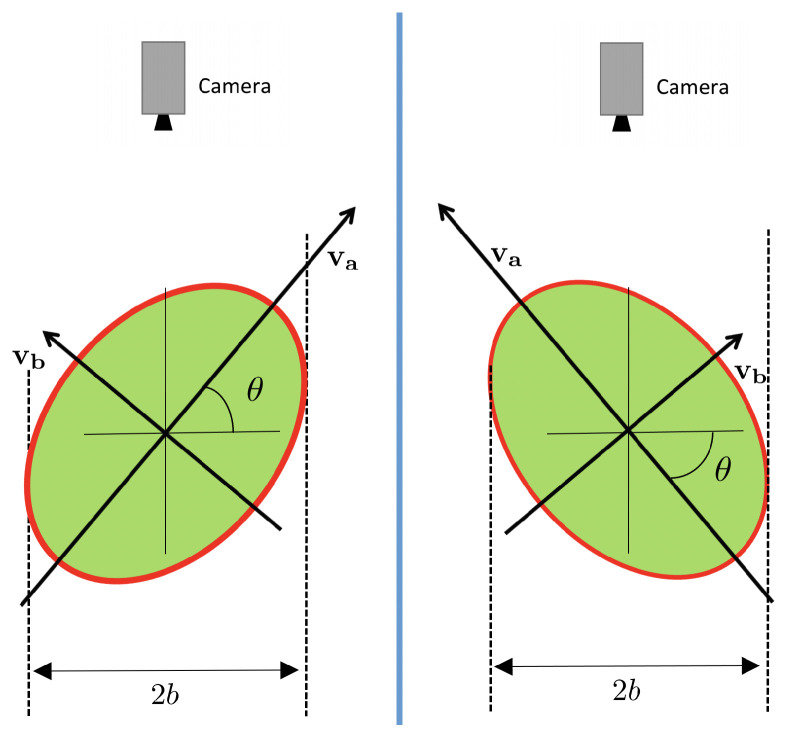
Ambiguity in the estimation of the elevation angle. The perceived shape from the camera is the same in both possibilities.

This equation allows for obtaining the elevation angle up to the ambiguity of the sign of θ. Figure 12 shows both possibilities. Fortunately, this ambiguity can easily be solved if the rotation direction of the fruit is known (as it always happens when using the roller conveyor machines that rotate the fruits in a known direction).

Consider the sequence $B=\{b_{i}\}$, $1<i<N_{v}$, created with the semi-minor axes of the projected ellipses of the different views.

**Figure 13:**
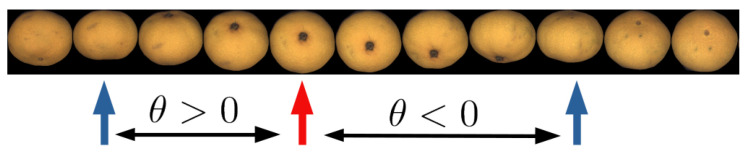
Sequence of views. Blue and red arrows indicate local minima and maxima respectively, of the sequence $B=\{b_{i}\}$ of the semi-minor axis. If the sequence B is increasing at instant *i*, then θ>0. This means (for this direction of rotation) that the part below the fruit center in the view is higher than the part above the center.

If the sequence B is increasing at $b_{i}$, i.e., $b_{i-1}<b_{i}<b_{i+1}$, and the fruit is rotating downwards as seen from the camera (see Figure 13), the elevation angle will be θ>0, meaning that the part below the center of the fruit has a greater height than that above the center. On the contrary, if the sequence is decreasing at $b_{i}$, the upper part will be above the fruit center. The same discussion applies if the fruit is known to be rotating upwards as seen from the camera, but with opposite results.

The local extrema of B correspond to elevation angles $\theta_{i}\approx 0$ or $\theta_{i}\approx \pm \pi /2$. Due to the symmetry, both possibilities of θ generate very similar *z*-coordinates and therefore are almost interchangeable. To solve the ambiguity in this case, the adopted solution is the one that generates a smoother sequence of θ values (i.e., the option with an angle $\theta_{i}$ closer to $\theta_{i-1}$ or $\theta_{i+1}$).

#### 3.1.4. Pixels 3D Coordinates

In order to estimate 3D rotations, the height *z* of every pixel is needed as explained in Section 3.2.

The case of spherical model is particularly simple. If *A* is the radius of the sphere, then the *z*-coordinate of pixel at image position (x,y) is:

(8) $$z=\sqrt{A^{2}-{\left(x-c_{x}\right)}^{2}-{\left(y-c_{y}\right)}^{2}}$$

where $\left(c_{x},c_{y}\right)$ is the center of the projected ellipse in the view (Equation (3)).

For non-spheric spheroids, the computation of *z* is a little bit more elaborate. For simplicity of the presentation, it will only be derived for an oblate spheroid. The equation of an axis-aligned oblate spheroid can be written as:

(9) $${\left(\frac{x}{B}\right)}^{2}+{\left(\frac{y}{A}\right)}^{2}+{\left(\frac{z}{A}\right)}^{2}=1$$

where *A* and *B* are the lengths of the semi-principal axes (A>B), or, equivalently, in matrix form as:

(10) $$x^{T}\left(\begin{array}{ccc} 1/B^{2} & 0 & 0\\ 0 & 1/A^{2} & 0\\ 0 & 0 & 1/A^{2} \end{array}\right)x=1$$

with $x^{T}=\left(x,y,z\right)$.

In general, spheroid axes are not aligned with respect to camera axes. It is necessary to introduce a pose matrix P. The rows of this matrix are the coordinates of the spheroid principal axes in the camera reference frame.

The elements of P are:

(11) $$P=\left(\begin{array}{ccc} p_{11} & p_{12} & p_{13}\\ p_{21} & p_{22} & p_{23}\\ p_{31} & p_{32} & p_{33} \end{array}\right)$$

and can be derived from the eigenvectors of the 2D covariance matrix Σ of the projected ellipse in each view and the elevation angle θ. Let $v_{1}=\left(v_{1x},v_{1y}\right)$ and $v_{2}=\left(v_{2x},v_{2y}\right)$ be the unit-length eigenvectors of Σ (see Figure 10).

In order to obtain the vector of the first (minor) axis of the spheroid, we need to compute the elevation angle, as described in Section 3.1.3. Assuming that we have already computed it, the first row of P is (unit vector in direction $v_{b}$ in Figure 11):

$$p_{11}=v_{2x}\;\sin\theta \;\;p_{12}=v_{2y}\;\sin\theta \;\;p_{13}=\cos\theta$$

The second axis of the spheroid (semi-axis length *A*) can be chosen aligned to $v_{1}$ and parallel to plane z=0:

$$p_{21}=v_{1x}\;\;p_{22}=v_{1y}\;\;p_{23}=0$$

The third row of the matrix can simply be obtained using the cross product of the first two rows:

$$\left(p_{31},p_{32},p_{33}\right)=\left(p_{11},p_{12},p_{13}\right)\times \left(p_{21},p_{22},p_{23}\right)$$

Therefore, the equation of a generic spheroid in a generic orientation position can be written as:

(12) $$x^{T}P^{T}\left(\begin{array}{ccc} 1/B^{2} & 0 & 0\\ 0 & 1/A^{2} & 0\\ 0 & 0 & 1/A^{2} \end{array}\right)Px=1$$

(13) $$x^{T}Ax=1$$

where

$$A=P^{T}\left(\begin{array}{ccc} 1/B^{2} & 0 & 0\\ 0 & 1/A^{2} & 0\\ 0 & 0 & 1/A^{2} \end{array}\right)P$$

Thus, given the centered coordinates, $\left(x^{\prime },y^{\prime }\right)$, of the pixel at image coordinates (x,y):

(14) $$\left(x^{\prime },y^{\prime }\right)=\left(x-c_{x},y-c_{y}\right)$$

the *z*-value can be obtained by solving the following second degree equation and keeping the largest solution (the one closer to +∞):

(15) $$\left(x^{\prime },y^{\prime },z\right)A\left(\begin{array}{c} x^{\prime }\\ y^{\prime }\\ z \end{array}\right)=1$$

Details about how to solve this equation are given in Appendix A.

### 3.2. 3D Rotation Estimation

This section explains how to estimate the 3D rotation between two consecutive views of the fruit. The rotation matrix R transforms the 3D coordinates of one point $p_{s}$ in the source view to the target view:

(16) $$p_{t}=R\;p_{s}.$$

The strategy to estimate the rotation between two consecutive views is to perform an exhaustive search in the space of feasible rotations and compute a cost measurement for each one. Then, the rotation with lowest cost is selected as the initial estimate.

The error function compares the transformed source and target images using a set of relevant points L. The main reason to use a set of relevant points instead of all the points from the source fruit is efficiency. Section 3.3.2 describes how the relevant points are selected in detail. Once the set of relevant points L is available, the 3D coordinates $p_{s}=\left(x_{s}^{\prime },y_{s}^{\prime },z_{s}\right)$ of every point $\left(x_{s},y_{s}\right)$, p∈L, are calculated by solving Equation (15).

Let $p_{t}=\left(x_{t}^{\prime },y_{t}^{\prime },z_{t}\right)$ be the coordinates in the target view of the transformed point $p_{s}$, which is obtained using a candidate rotation R:

- If $z_{t}<0$, the transformed point $p_{t}$ is not visible in the target image and it is ignored in the similarity computation.
- If $z_{t}>0$, then the pair $\left(p_{s},p_{t}\right)$ is added to a list S of valid relevant points.

Then, the error function for a candidate rotation R is then obtained as follows:

(17) $$\epsilon \left(R\right)=\frac{1}{\left|S\right|}\sum_{S}\left|Im_{s}\left(x_{s},y_{s}\right)-Im_{t}\left(x_{t},y_{t}\right)\right|$$

where $Im_{s}$ and $Im_{t}$ are the source and target images after some pre-processing described in Section 3.3.1. Finally, the estimated rotation between the two views is:

(18) $$\hat{R}=\underset{R\in R}{arg min}\left\{\epsilon (R)\right\}$$

where R is the set of plausible rotations.

Rotations in 3D can be described by the so-called *rotation vectors*, $\vec{v}=\vec{k}\theta$, where $\vec{k}$ is a unit vector that defines the rotation axis, and θ is the rotation angle around that axis. Using the Rodrigues’ Formula [33], it is possible to obtain the rotation matrix R from $\vec{v}$.

Although the rotation vector $\vec{v}=\left(r_{x},r_{y},r_{z}\right)$ has three components, our empirical experiments showed that the $r_{z}$ component is negligible ($r_{z}\approx 0$), i.e., the rotation vector lies on the XY plane.

Since the rollers of the conveyor belt force the fruits to rotate around the *x*-axis (Figure 2), the largest component of a possible $\vec{v}$ is $r_{x}$. The component $r_{y}$ should also be close to zero for ideal shaped fruits. However, the unavoidable imperfections of real fruits result in $r_{y}$ possibly being non-zero.

**Figure 14:**
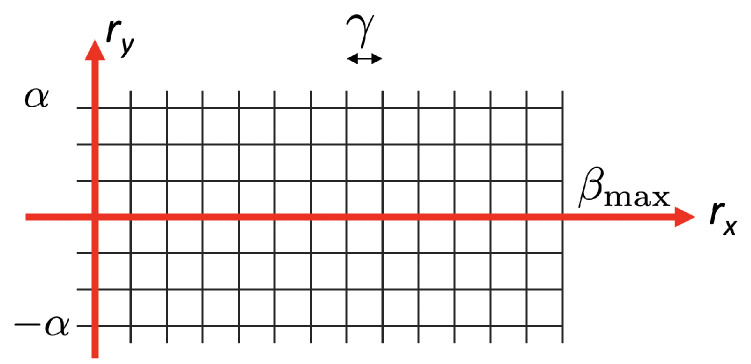
Search grid of rotation vectors.

These constraints define the set R of plausible rotations for the search, so that rotation vectors $\vec{v}$ (and consequently candidate matrices R) are sampled from a 2D grid on the $\left(r_{x},r_{y}\right)$ space. Figure 14 shows the search space, where $r_{x}$ is in the range 0–$\beta_{\max}$, where $\beta_{\max}$ is the maximum expected rotation that is determined by the mechanic setup, and $r_{y}$ is between −α and α (typically, we use α=10 degrees). The grid is sampled with a step γ=1 degrees (configurable).

**Figure 15:**
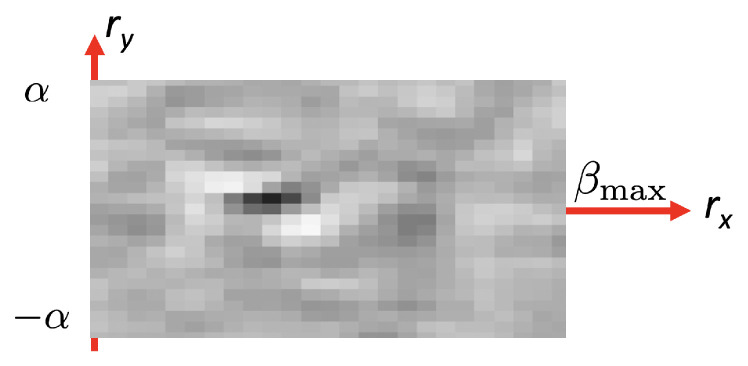
Error map for all rotations in the grid search.

The values of ϵ(R) can also be represented as an *error map* that represents the obtained error for each point $\left(r_{x},r_{y}\right)$ of the search grid of Figure 14. Figure 15 shows an example of one error map, where the initial estimate of the rotation, $\hat{R}$, is located at the position of the darkest pixel in that error map.

**Figure 16:**
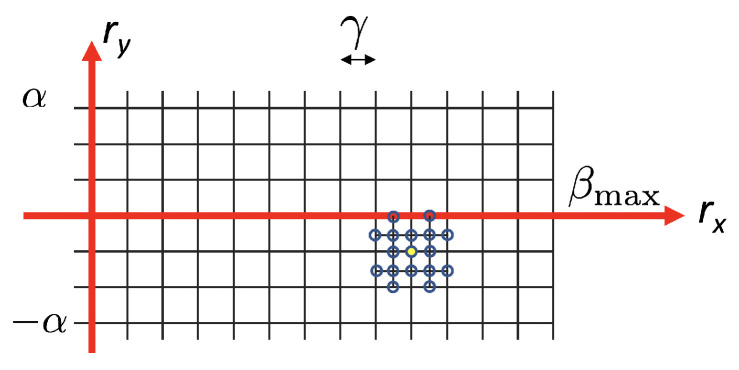
This figure illustrates the local increase in resolution of the error map around the local minimum. The initial local minimum obtained with γ step is shown as a yellow filled circle.

However, this initial estimate is relatively coarse due to the γ quantization step of the search grid. In order to refine this initial estimate, two operations are performed:

- New intermediate rotations, with step γ/2, are computed around the local minimum $\hat{R}$. The new sampled rotations are shown as empty circles in Figure 16. Then, the minimum on this denser 5×5 subgrid is found.
- A parabola is fitted locally around the new minimum. The final rotation is obtained as the position of the parabola minimum. This idea is similar to that proposed by Lowe for local extrema detection in SIFT [34]. More details about this parabolic refinement are given in Appendix B.

### 3.3. Implementation Details

This section presents some implementation details which are needed to estimate the fruit rotations in real time in a production scenario.

#### 3.3.1. Image Pre-Processing

The standard fruit images (as those found in Figure 6) are RGB images with a spatial resolution of about 75 pix/inch (that translates to image sizes in the range of 200–350 pixels width/height).

The goal of image pre-processing is to obtain good smaller images to compare source and target views and to build a small list of relevant points, L, to estimate the rotation (Section 3.2). The following operations are performed:

1. Reduce the number of color channels. Unlike many approaches, this step is accomplished by simply taking the green component. Compared with standard RGB to luminance conversion, taking the green component is computationally free.
2. Reduce the image resolution. In our experiments, we use a downsampling factor of 4 in both axes. To mitigate aliasing, each pixel of the downsized image is computed using the average of the corresponding 4×4 block in the original image.
3. High pass filtering. This step is performed by computing the signed difference between the downsampled image and a Gaussian blurred version of it with σ=1.25. This image will be zero at smooth portions of the fruit and will exhibit large positive or negative values at details or texture. A fast recursive and separable implementation of the Gaussian filter was used [35].

**Figure 17:**
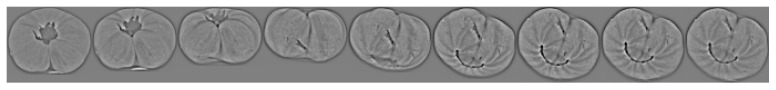
Result of image pre-processing.

Figure 17 shows an example of the result of the image pre-processing of the views in Figure 3. Notice that this high-pass image is computed at a resolution 4×4 smaller than the original input image, allowing the computation to be $4^{2}=16$ times faster.

#### 3.3.2. Selecting Relevant Points

The proposed algorithm to estimate the 3D rotation between two views of a fruit can be computationally expensive due to the exhaustive search in the space of possible rotations R.

Remember that, for each possible rotation R∈R, a certain set of source points L must be mapped to the target using one 3×3 matrix multiplication by a 3×1 vector.

This is a very high time-consuming operation. In order to accelerate the computation time, a list of relevant source points L is created, as introduced in Section 3.2. This section presents the details about how L is created.

**Figure 18:**
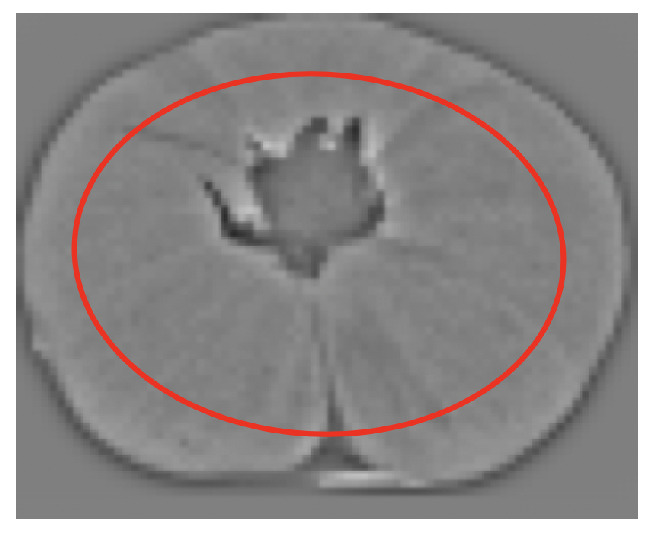
Area from which relevant points are obtained.

Let $Im_{s}$ be a pre-processed source image as shown in Figure 18. The first constraint is that points near the fruit border will be discarded (outside the red ellipse in Figure 18). These points are not interesting because, when rotated in 3D, they may not be visible in the target view. The size of this red ellipse depends on the maximum expected rotation.

Then, the points selected are those above the 97th percentile of the absolute value inside the red ellipse.

Using the previous settings, the typical number of points in L lies in the range from 50 to 100 points per source image. The precise number depends on the image size and the fraction of retained points.

### 3.4. Datasets

This section presents the two datasets that have been used to evaluate the performance of the proposed method to estimate the 3D rotation of fruits.

#### 3.4.1. FruitRot3D Dataset

**Figure 19:**
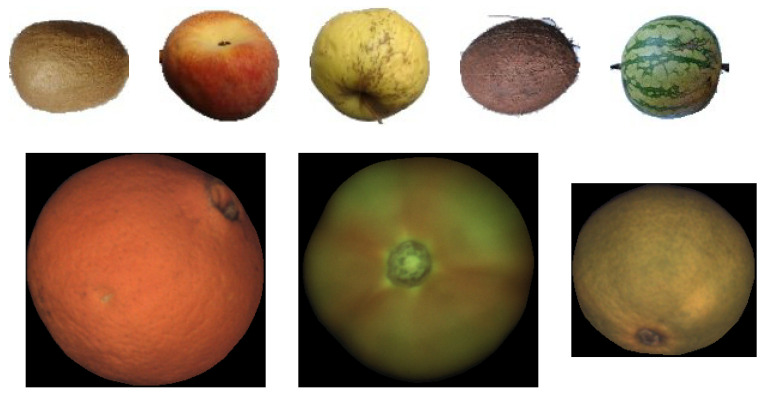
**Top**: Sample Images from Fruits-360 data set; from left to right kiwi, peach, apple golden, coconut, and watermelon; **Bottom**: Sample Images from FruitRot3D dataset; from left to right, orange, tomato, and mandarin.

The images in this dataset were captured using an industrial fruit inspection machine with a roller conveyor unit that simulates real operation conditions using diffuse illumination to prevent potential highlights. The dataset has been made publicly available to the community so that the results of this work can be replicated [36]. Figure 19-bottom shows a few samples of images of this dataset.

A few key aspects of this dataset are:

- The dataset contains three types of fruits, namely oranges, mandarins, and tomatoes.
- There are 15 fruit sequences for each fruit type.
- The length of each fruit sequence oscillates between 13 and 16 images.
- The 3D rotation between consecutive views of the same fruit is not constant due to the slipping on the rolling conveyor and irregularities on the fruit shape. Notice that not only the magnitude of the rotation can change but also its axis. The typical range of the magnitude of the rotation is between 10 and 30 degrees.
- The Foreground/Background segmentation was automatically performed by the inspection machine. Background pixels were set to black (RGB = {0,0,0}).
- The imaged fruit diameters are in the range between 250 and 350 pixels depending on the fruit type.
- The images are stored in PNG format with lossless compression.

#### 3.4.2. Fruits-360 Dataset

This dataset was originally intended to train machine learning models to recognize fruits from different view angles [37]. In order to easily create many training samples, fruits and vegetables were planted in the shaft of a low speed motor (3 rpm) and a short movie of 20 s was recorded using a Logitech C920 camera (Lausanne, Switzerland). A white sheet of paper was placed as a background so that fruits could be easily segmented.

Some aspects of this dataset are:

- The dataset contains more than 100 types of fruits. However, only one sequence per fruit type is available.
- If it is assumed that both the webcam frame rate (no image drops) and the motor speed are constant, then the 3D rotation between consecutive views must be constant. Using these assumptions, the approximate rotation magnitude between consecutive images is about one degree.
- The images in the dataset were resized to a fixed common size 100×100 pixels.
- The images were stored using JPEG lossy compression.

The Fruits-360 dataset is relevant in this work because it provides fruit sequences with controlled rotation. Although the exact magnitude of the rotation is not known, it can be assumed that both rotation axis and magnitude are constant and therefore objective measurements about the accuracy of the proposed method can be made. Another interesting feature of this dataset is that it contains many different fruit types. In this work, coconuts, kiwis, apples, peaches, and watermelons were selected to evaluate the proposed method. These fruits were selected because they have a textured surface and represent the three spheroid models: spherical, oblate, and prolate. Figure 19-top shows a few samples of images of this dataset.

## 4. Results

In this section, the performance of the proposed method for 3D rotation estimation is presented with three different experiments.

In Section 4.1, the rotation error is estimated in a controlled environment where the rotation speed of the fruits is kept constant.

In a real working scenario, it is not possible to accurately measure the rotation of the fruits (magnitude and axis). For this reason, the reprojection error is used in Section 4.2 to indirectly evaluate the performance of the algorithm in a more realistic scenario.

Finally, qualitative results that show how points can be tracked in fruit sequences are presented in Section 4.3

### 4.1. Rotation Error Analysis

In order to measure the rotation error of the proposed algorithm, the true rotation angle between consecutive views of each fruit should be known. Unfortunately, no practical method was found to obtain these ground-truth rotations in a real inspection machine. Although some preliminary experiments were performed with tennis balls and marked fruits, these experiments did not fully resemble the real working conditions. In the case of tennis balls, the geometry fits better the spherical model than any real fruit. In the case of marked fruits, the presence of the markings makes the estimation of the rotation simpler than when real fruits are used.

To overcome this problem, the Fruits-360 dataset that contains sequences of rotating fruits in a controlled environment was selected to measure the rotation error. The rotation speed in the Fruits-360 dataset can be assumed to be constant but unknown. In this dataset, the rotation between consecutive views *n* and n+1 is very small (around one degree), and much smaller than the rotation angle in real inspection machines (15–30 degrees). For this reason, rotations between views *n* and n+Δn were estimated.

**Figure 20:**
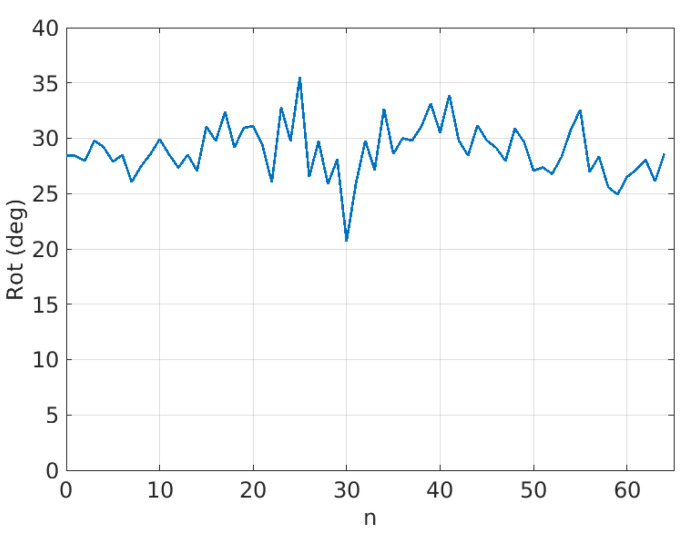
Sequence of estimated rotations for the coconut sequence with Δn=20.

Figure 20 shows an example of estimated rotations for the coconut sequence with Δn=20.

**Figure 21:**
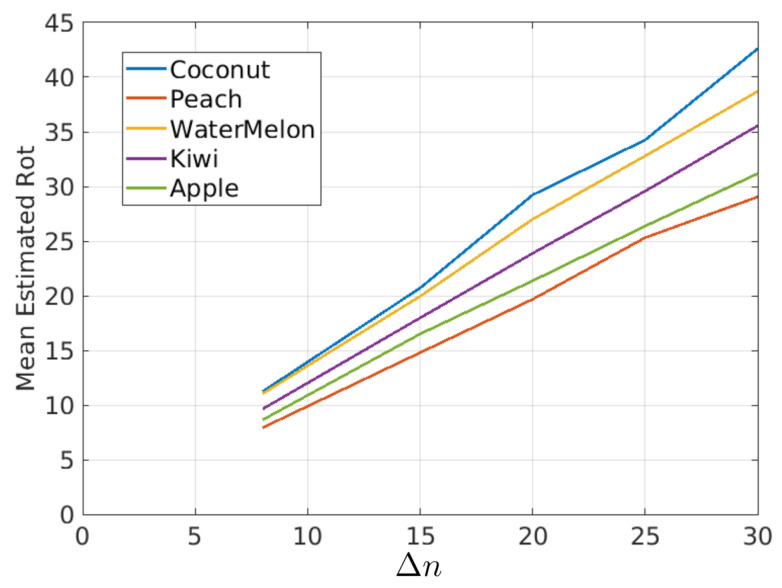
Mean Rotations as a function of Δn for different fruit types.

**Figure 22:**
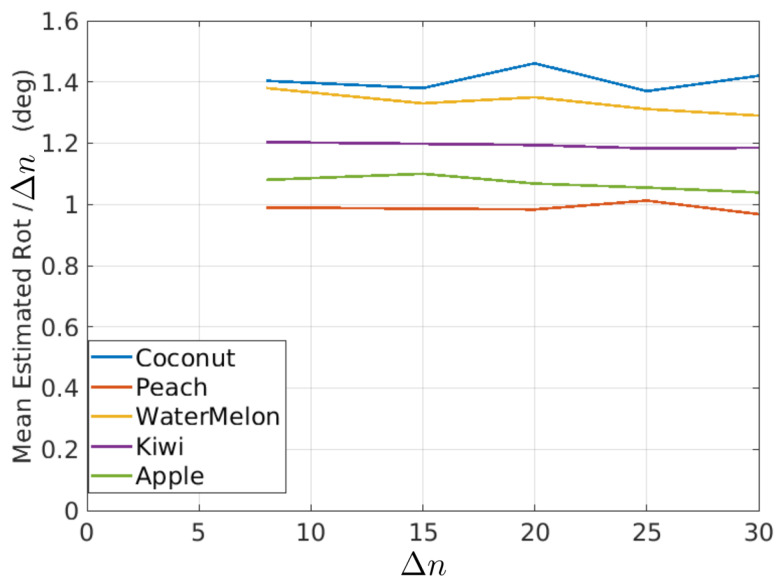
Mean rotation speed as a function of Δn for different fruit types.

In this figure, it is possible to observe that the estimated rotations can be modeled as a mean value plus some random variations. The random variations should ideally be zero and can be described with the variance (or standard deviation) of the estimated rotations. On the other hand, if it is assumed that the true rotation speed is constant, then the mean value of the rotations in Figure 20 should be proportional to Δn as shown in Figure 21 and Table 1, where the mean of estimated rotations as a function of Δn are shown for the coconut, peach, watermelon, kiwi, and apple sequences of the Fruits-360 dataset. Figure 22 and Table 2 show the rotation speeds calculated as mean_rotation/Δn. This figure shows that the estimated rotations are consistent with the data obtained by rotating fruits at constant speed.

**Figure 23:**
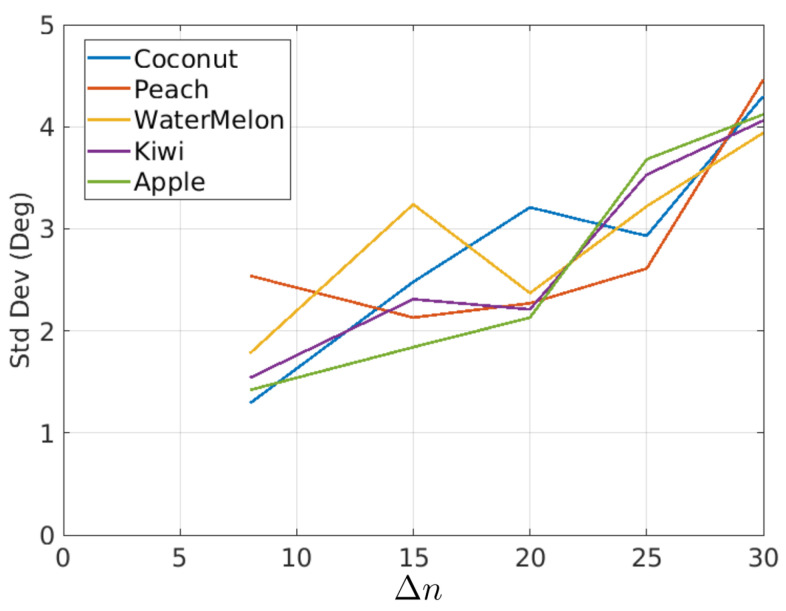
Standard deviation of rotations as a function of Δn for different fruit types.

In Figure 20, it was shown that the rotations had some variations around their mean value. The standard deviation is a measure of such variations. Figure 23 and Table 3 show the standard deviation as a function of Δn and fruit type.

Several conclusions can be obtained from this experiment using fruits that rotate in a controlled manner:

- The proposed method seems to work well for a relatively broad range of fruit types. The only restriction is the presence of texture and a reasonable similarity to the geometric model.
- Typically, industrial inspection machines are adjusted for rotations between consecutive views in the range 18–30 degrees. The method provides consistent rotation estimates for rotations in that range. This fact can be derived from Figure 22 and Table 2 where the average rotation speed is almost constant regardless of the Δn value.
- Figure 23 shows that standard deviation increases as Δn increases. The reason for this is that the overlapped area of views decreases as the inter-view rotation increases yielding a noisier error map (Figure 15).

### 4.2. Reprojection Error Analysis

An indirect way to measure the goodness of the estimated rotations is to compare where a point in view *i* would be mapped in view i±1 using the estimated rotation and compare it with its *ground-truth* position annotated by a human.

**Figure 24:**
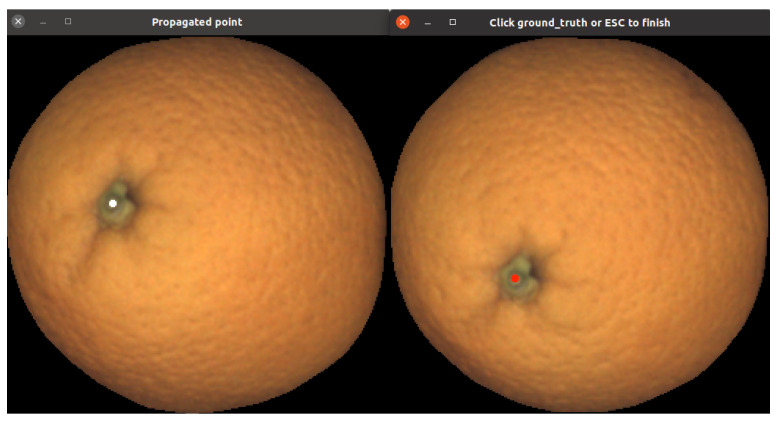
This figure illustrates the idea of the interface to annotate ground-truth for estimating reprojection error. The user is requested to select corresponding points in both views.

To ease the ground-truthing task, an interface that allows a user to select point correspondences between consecutive views was developed. Figure 24 shows the interface; the user must click point correspondences in both views. These points allow for obtaining the rms error between the automatically predicted position and the ground-truth point.

The reprojection errors were estimated using the FruitRot3D dataset. The reason is that the image resolution in the Fruits-360 dataset is very small, and it is really difficult to establish point correspondences even for a human annotator.

Overall, about 200 point correspondences per fruit class were used to evaluate the reprojection error. The results are summarized in Table 4. Since the reprojection error is measured in pixels and its magnitude depends on the image resolution, Table 4 shows the rms error in pixels and also relative to the fruit diameter.

One first observation is that oblate fruits tend to have larger errors than spherical ones. This may be due to the fact that the spherical model fits better actual fruits than oblate ones. In addition, non-spheric fruits need to estimate the elevation angle θ to obtain the *z*-coordinate (Section 3.1.4) of the point. Errors in the estimation of elevation angle θ increase the error in the estimated *z*, which in turn increases the total reprojection error.

Another important consideration is that a non-negligible part of the observed error is due to human annotation error itself since real fruits have no clear landmarks that can be identified within less than a few pixels accuracy.

The ground-truth point correspondences used in this experiment have been made public in [36].

### 4.3. Point Tracking along a Sequence of Views

This section provides qualitative results by showing how a point selected in one view can be tracked along the sequence of views using the estimated rotations between consecutive views. This experiment is useful to figure out how the method would perform to prevent multiple counts of the same defect.

In the examples in this section, a point is manually selected in one view and its position predicted in the other views. To do so, the 3D coordinates of the initial point are obtained from the fitted geometric model (see Section 3.1.4), and then the estimated 3D rotations are applied to it. In order to propagate beyond adjacent views, 3D rotations are concatenated by multiplying the corresponding rotation matrices. For backward propagation, the inverse of the rotation matrix is used.

**Figure 25:**
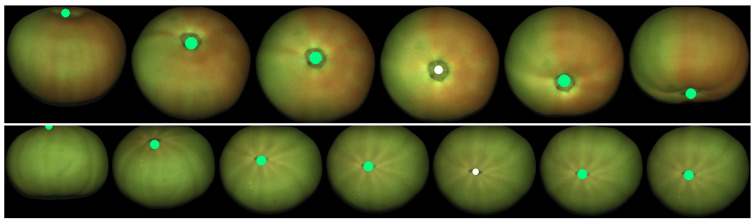
Example of point tracking in the case of two different tomatoes. The initial tracked point is white. Green circles mean predicted visible positions. The geometry model is set to oblate in this case. The sequence has been truncated to the views where the tracked point remains visible.

**Figure 26:**
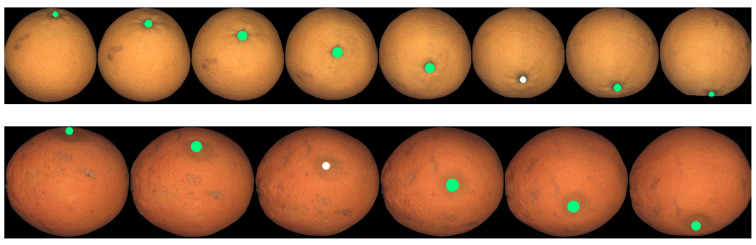
Example of point tracking in the case of two different oranges. The geometry model in this case is sphere. The sequence has been truncated to the views where the tracked point remains visible.

**Figure 27:**
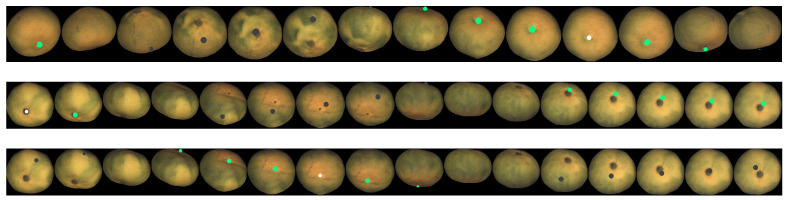
Example of point tracking in the case of three different mandarins. Dark circles mean predicted occluded positions of the initial point. The third row is the same fruit as the second, but a different point is tracked. Oblate geometry has been used.

**Figure 28:**
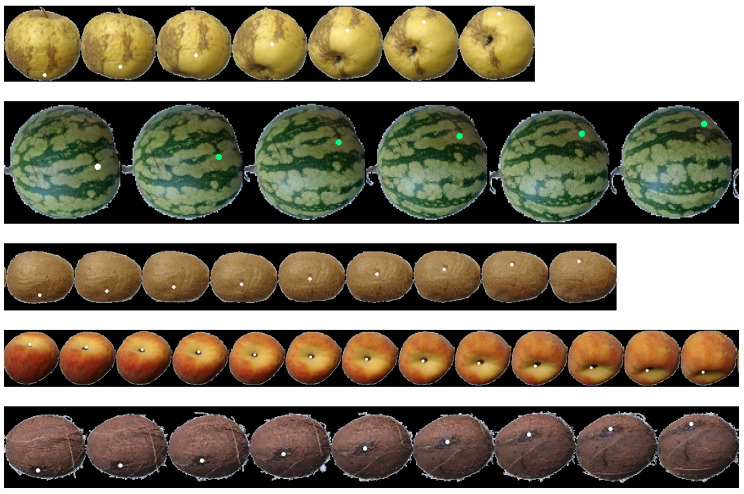
Examples of point tracking of fruits in the Fruit-360 dataset.

This point tracking has been applied to fruits from both datasets. Figure 25, Figure 26 and Figure 27 show some tracking results of the FruitRot3D dataset. The precision of the whole process can be observed in these figures. Two interesting examples can be seen in the two first rows of Figure 27, where the tracked point reappears after completing a full 360-degree rotation. In this figure, the tracked points are highlighted in green color if the tracked point is visible; otherwise, it is highlighted using a dark color to emphasize that it is not visible in that view (it lies on the hidden side). These qualitative examples are quite remarkable because they show that, despite many consecutive rotations being used, it is still possible to predict with relative precision where the point reappears after occlusion. Qualitative results using the Fruits-360 dataset are also provided in Figure 28. From the observation of Figure 25, Figure 26, Figure 27 and Figure 28, some conclusions can be drawn:

- No bias is observed. If the estimated rotations were biased, then a drift in the predicted position of tracked points would be observed.
- From Figure 28, it can be seen that the geometric model is relatively robust to imperfections in the foreground/background segmentation. The presence of stems (watermelon) or noisy contours (coconuts) did not affect the ability of the method to track points.
- The tracking precision is enough for pairing defects across views and prevent multiple counting of the same defect.
- The method has proved its applicability to very different kinds of fruits. The only limitations are that the geometry of the fruit can be reasonably modeled by a spheroid and the fruit skin contains enough texture variations.

## 5. Conclusions 

In this paper, a method to estimate the 3D rotation between pairs of consecutive views of fruits has been presented.

The key idea is to fit a 3D spheroid model for the fruit, and then estimate the 3D rotation using an exhaustive search in a small space of feasible rotations. Parabolic refinement is also used to increase the accuracy of the estimations.

In order to estimate the matching error, each candidate rotation R∈R, is applied to a very small number of points L of the source image. The use of a small set of points instead of all the points (as it is normally done in block-matching motion estimation) is the most important idea to boost the processing speed.

The algorithm has been tested with several types of fruits from two data-sets, one obtained with a real inspection machine and another one where controlled rotation had been applied to fruits.

The FruitRot3D data-set has been made public and can be freely used for prospective researchers in the field.

Although the spheroid model may seem too simplistic, the estimated rotations are very precise and allow for tracking points in the surface of the fruits along all the views.

In the context of fruit inspection, the proposed method can be used to assess if the whole fruit surface has been observed and also to track surface defects and prevent counting them more than once.

Special attention has been given to speed up all the computations. The whole process, including geometry, pose modeling, and the rotation estimation itself, can be done in less than 0.5 ms per view on a standard PC using one single core (year 2018, Intel I7@4 GHz).

Future research will use the estimated 3D rotations to *unroll* the fruit surfaces on a 2D topographic map so that fruit skin can be easily analyzed as a single whole.

## Figures and Tables

**Table 1:** Mean rotations for different values of Δn and fruit type. Data corresponding to curves of Figure 21.

	Coconut	Peach	Watermelon	Kiwi	Apple
	Prolate	Oblate	Spherical	Prolate	Oblate
Δn=8	11.23	7.92	11.0400	9.63	8.60
Δn=15	20.70	14.79	19.95	17.97	16.49
Δn=20	29.22	19.67	27.01	23.88	21.36
Δn=25	34.25	25.31	32.79	29.57	26.37
Δn=30	42.60	29.04	38.72	35.55	31.17

**Table 2:** Mean rotation speed for different values of Δn and fruit type. Data corresponding to curves of Figure 22.

	Coconut	Peach	Watermelon	Kiwi	Apple
Δn=8	1.40	0.99	1.38	1.20	1.08
Δn=15	1.38	0.98	1.33	1.19	1.10
Δn=20	1.46	0.98	1.35	1.19	1.06
Δn=25	1.37	1.01	1.31	1.18	1.05
Δn=30	1.42	0.96	1.29	1.18	1.03

**Table 3:** Standard deviation of estimated rotations as a function of Δn and fruit type, data corresponding to curves of Figure 23.

	Coconut	Peach	Watermelon	Kiwi	Apple
Δn=8	1.29	2.54	1.78	1.54	1.42
Δn=15	2.48	2.13	3.24	2.31	1.84
Δn=20	3.21	2.27	2.37	2.21	2.13
Δn=25	2.93	2.61	3.22	3.53	3.68
Δn=30	4.30	4.46	3.94	4.06	4.12

**Table 4:** RMS reprojection-error for different kinds of fruits.

Fruit Type	RMS-Error (Pixels)	RMS-Error/Diameter(%)
Oranges	2.53	0.94%
Mandarins	6.5	2.99%
Tomatoes	5.2	3.14%

## Data Availability

Datasets used are available at https://github.com/alalbiol/3d-rotation-estimation-fruits (accessed on 15 January 2021) and https://github.com/Horea94/Fruit-Images-Dataset (accessed on 9 September 2020).

## Appendix A. Solution of Quadratic Equation to Compute the Z-Coordinate of a Pixel

This section presents how to obtain a explicit solution for *Z* in the equation:

(A1) $$\left(x^{\prime },y^{\prime },Z\right)A\left(\begin{array}{c} x^{\prime }\\ y^{\prime }\\ Z \end{array}\right)=1$$

Consider the A matrix elements:

(A2) $$A=\left(\begin{array}{ccc} a_{11} & a_{12} & a_{13}\\ a_{21} & a_{22} & a_{23}\\ a_{31} & a_{32} & a_{33} \end{array}\right)$$

Equation (A1) can be expanded as:

(A3) $$\left(x^{\prime },y^{\prime }\right)\left(\begin{array}{cc} a_{11} & a_{12}\\ a_{21} & a_{22} \end{array}\right)\left(\begin{array}{c} x^{\prime }\\ y^{\prime } \end{array}\right)+\left(x^{\prime },y^{\prime }\right)\left(\begin{array}{c} a_{13}\\ a_{23} \end{array}\right)Z+\left(a_{31},a_{32}\right)\left(\begin{array}{c} x^{\prime }\\ y^{\prime } \end{array}\right)Z+a_{33}Z^{2}=1$$

Calling

$$C=\left(x^{\prime },y^{\prime }\right)\left(\begin{array}{cc} a_{11} & a_{12}\\ a_{21} & a_{22} \end{array}\right)\left(\begin{array}{c} x^{\prime }\\ y^{\prime } \end{array}\right)-1$$

and

$$B=\left(x^{\prime },y^{\prime }\right)\left(\begin{array}{c} a_{13}\\ a_{23} \end{array}\right)+\left(a_{31},a_{32}\right)\left(\begin{array}{c} x^{\prime }\\ y^{\prime } \end{array}\right)$$

allows for rewriting Equation (A3) as:

(A4) $$a_{33}Z^{2}+B\;Z+C=0$$

where *B* and *C* are scalar constants. The solution to this equation comes from the well known formula for quadratic equations:

$$Z=\frac{-B\pm \sqrt{B^{2}-4a_{33}C}}{2a_{33}}$$

The *Z*-coordinate of the pixel will be the solution closer to +∞, namely:

(A5) $$Z=\frac{-B+\sqrt{B^{2}-4a_{33}C}}{2a_{33}}$$

## Appendix B. Quadratic Refine

The Taylor expansion of a two-dimensional function up to a quadratic term around the origin has the form:

(A6) $$D\left(x\right)=D+\frac{\partial D^{T}}{\partial x}\;x+\frac{1}{2}x^{T}\;\frac{\partial^{2}D}{\partial x^{2}}\;x$$

where $\frac{\partial D}{\partial x}$ is the gradient vector:

$$\frac{\partial D}{\partial x}={\left(\frac{\partial D}{\partial x},\frac{\partial D}{\partial y}\right)}^{T}$$

and $\frac{\partial^{2}D}{\partial x^{2}}$ is the Hessian matrix:

$$\left(\begin{array}{cc} \frac{\partial^{2}D}{\partial x^{2}} & \frac{\partial^{2}D}{\partial x\partial y}\\ \frac{\partial^{2}D}{\partial x\partial y} & \frac{\partial^{2}D}{\partial y^{2}} \end{array}\right)$$

Computing the gradient of this expression and setting it to zero allow for finding the local minimum of the quadratic approximation:

(A7) $$\hat{x}=-{\left(\frac{\partial^{2}D}{\partial x^{2}}\right)}^{-1}\;\frac{\partial D}{\partial x}$$

The values of the Hessian matrix and gradient vector in Equation (A6) can be approximated as follows. For the horizontal gradient, the horizontal centered difference is computed

$$s_{x}\left(x,y\right)=\frac{D(x+1,y)-D(x-1,y)}{2}$$

and then a vertical weighted average is computed:

$$\frac{\partial D}{\partial x}\approx D_{x}\left(x,y\right)=\frac{s_{x}\left(x,y+1\right)+2s_{x}\left(x,y\right)+s_{x}\left(x,y-1\right)}{4}$$

Similarly, the vertical component of the gradient is approximated as:

$$s_{y}\left(x,y\right)=\frac{D(x,y+1)-D(x,y-1)}{2}$$

$$\frac{\partial D}{\partial y}\approx D_{y}\left(x,y\right)=\frac{s_{y}\left(x+1,y\right)+2s_{y}\left(x,y\right)+s_{y}\left(x-1,y\right)}{4}$$

The second derivatives are estimated as:

$$s_{xx}\left(x,y\right)=D\left(x+1,y\right)-2\;D\left(x,y\right)+D\left(x-1,y\right)$$

$$\frac{\partial^{2}D}{\partial x^{2}}\approx D_{xx}\left(x,y\right)=\frac{s_{xx}\left(x,y+1\right)+2s_{xx}\left(x,y\right)+s_{xx}\left(x,y-1\right)}{4}$$

$$s_{yy}\left(x,y\right)=D\left(x,y+1\right)-2\;D\left(x,y\right)+D\left(x,y-1\right)$$

$$\frac{\partial^{2}D}{\partial y^{2}}\approx D_{yy}\left(x,y\right)=\frac{s_{yy}\left(x+1,y\right)+2s_{yy}\left(x,y\right)+s_{yy}\left(x-1,y\right)}{4}$$

The second order mixed partial derivatives will be approximated as:

$$\frac{\partial^{2}D}{\partial x\partial y}\approx D_{xy}\left(x,y\right)=\frac{D(x+1,y+1)+D(x-1,y-1)-D(x-1,y+1)-D(x+1,y-1)}{4}$$

Once the gradient vector and the Hessian matrix have been approximated using the previous equations, the minimum of the quadratic approximation is obtained using Equation (A7).
